# Expression and characterization of highly antigenic domains of chicken anemia virus viral VP2 and VP3 subunit proteins in a recombinant *E. coli* for sero-diagnostic applications

**DOI:** 10.1186/1746-6148-9-161

**Published:** 2013-08-13

**Authors:** Guan-Hua Lai, Ming-Kuem Lin, Yi-Yang Lien, Jiun-Hau Fu, Hsi-Jien Chen, Chi-Hung Huang, Jason TC Tzen, Meng-Shiou Lee

**Affiliations:** 1 Graduate Institute of Biotechnology, National Chung Hsing University, Taichung, Taiwan; 2 Department of Chinese Pharmaceutical Science and Chinese Medicine Resources, China Medical University, Taichung, Taiwan; 3 Department of Veterinary Medicine, National Pingtung University of Science and Technology, Pingtung, Taiwan; 4 Department of Safety, Health and Environmental Engineering, Mingchi University of Technology, Taipei, Taiwan; 5 Graduate School of Biotechnology, Hung kuang University, Taichung, Taiwan

## Abstract

**Background:**

Chicken anemia virus (CAV) is an important viral pathogen that causes anemia and severe immunodeficiency syndrome in chickens worldwide. Generally, CAV infection occurs via vertical transmission in young chicks that are less than two weeks old, which are very susceptible to the disease. Therefore, epidemiological investigations of CAV infection and/or the evaluation of the immunization status of chickens is necessary for disease control. Up to the present, systematically assessing viral protein antigenicity and/or determining the immunorelevant domain(s) of viral proteins during serological testing for CAV infection has never been performed. The expression, production and antigenic characterization of CAV viral proteins such as VP1, VP2 and VP3, and their use in the development of diagnostic kit would be useful for CAV infection prevention.

**Results:**

Three CAV viral proteins VP1, VP2 and VP3 was separately cloned and expressed in recombinant *E. coli*. The purified recombinant CAV VP1, VP2 and VP3 proteins were then used as antigens in order to evaluate their reactivity against chicken sera using indirect ELISA. The results indicated that VP2 and VP3 show good immunoreactivity with CAV-positive chicken sera, whereas VP1 was found to show less immunoreactivity than VP2 and VP3. To carry out the further antigenic characterization of the immunorelevant domains of the VP2 and VP3 proteins, five recombinant VP2 subunit proteins (VP2-435N, VP2-396N, VP2-345N, VP2-171C and VP2-318C) and three recombinant VP3 subunit proteins (VP3-123N, VP3-246M, VP3-366C), spanning the defined regions of VP2 and VP3 were separately produced by an *E. coli* expression system. These peptides were then used as antigens in indirect ELISAs against chicken sera. The results of these ELISAs using truncated recombinant VP2 and VP3 subunit proteins as coating antigen showed that VP2-345N, VP2-396N and VP3-246M gave good immunoreactivity with CAV-positive chicken sera compared to the other subunit proteins. Moreover, the VP2-396N and VP2-345 based ELISAs had better sensitivity (97.5%) and excellent specificity (100%) during serodiagnosis testing using a mean plus three standard deviations cut-off. The VP3-246M based ELISA showed a sensitivity of 85% and a specificity of 100% at the same cut-off value.

**Conclusions:**

This is the first report to systematically assess the antigenic characteristics of CAV viral proteins for sero-diagnosis purposes. Purified recombinant VP2-396N and VP2-345N subunit proteins, which span defined regions of VP2, were demonstrated to have good antigenicity and higher sensitivities than VP3-246M and were able to recognize CAV-positive chicken serum using an ELISA assay. The defined antigenicity potential of these chimeric subunit proteins produced by expression in *E. coli* seem to have potential and could be useful in the future for the development of the CAV diagnostic tests based on a subunit protein ELISA system.

## Background

Chicken anemia virus (CAV) is the member of the genus *Gyrovirus* of the family *Circoviridae*, and the genome consists of a circular single-stranded 2.3 kb DNA molecule [1]. CAV was first isolated in 1979 in Japan and is the major agent responsible for a disease causing severe anemia and immunosuppression [2]. The characteristic symptoms of the disease include aplasia of the bone marrow and the destruction of T lymphoid tissue, which has been shown histopathologically after CAV infection [3,4]. Generally, CAV as the causative agent of chicken anemia disease affects one-day old chicks that lack maternal antibodies [5]. Mortalities as high as 55% and morbidities as high as 80% have been described when chicks are infected with CAV [6].

The CAV virion is an icosahedral, nonenveloped and 18 nm diameter particle [1]. The 2.3 kb CAV genome encodes three viral proteins, VP1, VP2 and VP3 [1,2]. The VP1 protein is the sole structural protein assembled into the CAV capsid and has a size of 51 kDa [1]. VP2 is a 24 kDa protein that has phosphatase activity with dual specificity [7]. VP3 is a 13 kDa protein that shows apoptotic activity and is able to induces apoptosis within infected chicken cells and human tumor cell lines [8]. Immunogenicity studies have shown that VP1 and VP2 are crucial components for the elicitation of host-produced virus neutralizing antibodies in chickens [9]. Therefore, VP1 and VP2 have previously been thought to be good candidates for use as immunogens when developing subunit vaccines or diagnostic kits [9,10]. Up to the present, several different systems have been developed to express the three CAV viral proteins for use in serological tests or for the development of diagnostic ELISA kits to detect the presence of CAV antibodies [11-15]. In addition, VP2 and VP3 have been applied as target antigen to generate diagnostic monoclonal antibodies for immunological characterization and for the development of CAV detection kits [16,17]. To successfully develop the above areas, the further antigenic characterization of the CAV viral proteins is required if a useful diagnostic kit is to be developed. However, so far, the VP1, VP2 and VP3 proteins of CAV have never been used as antigens to assess comparatively their antigenicity against chicken sera.

In this study, we employed recombinant VP1, VP2 and VP3 proteins from CAV that were produced using *E. coli* to characterize their antigenicity with respect to chicken sera; the aim was to assess their usefulness for their possible immunological applications. Herein, recombinant VP2 and VP3 proteins were able to be recognized as having high antigenicity using clinical sera samples infected with CAV. In addition, various recombinant VP2 and VP3 truncated subunit proteins were also produced using *E. coli* and these were then systemically assessed for their antigenicity with respect to chicken sera; the aim being to evaluate the two proteins for potential immunorelevant domains. Finally, the productivities of three of the VP2 and VP3 subunit proteins, namely VP2-345N, VP2-396N and VP3-246M, were evaluated and compared. Using these findings, it was possible to create a VP2 subunit based ELISA that had high specificity and sensitivity; this has the potential to become a valuable immunological tool for the detection of CAV infection.

## Results

### Expression, purification and antigenic characterization of the CAV VP1, VP2 and VP3 proteins in an *E. coli* expression system

**Figure 1:**
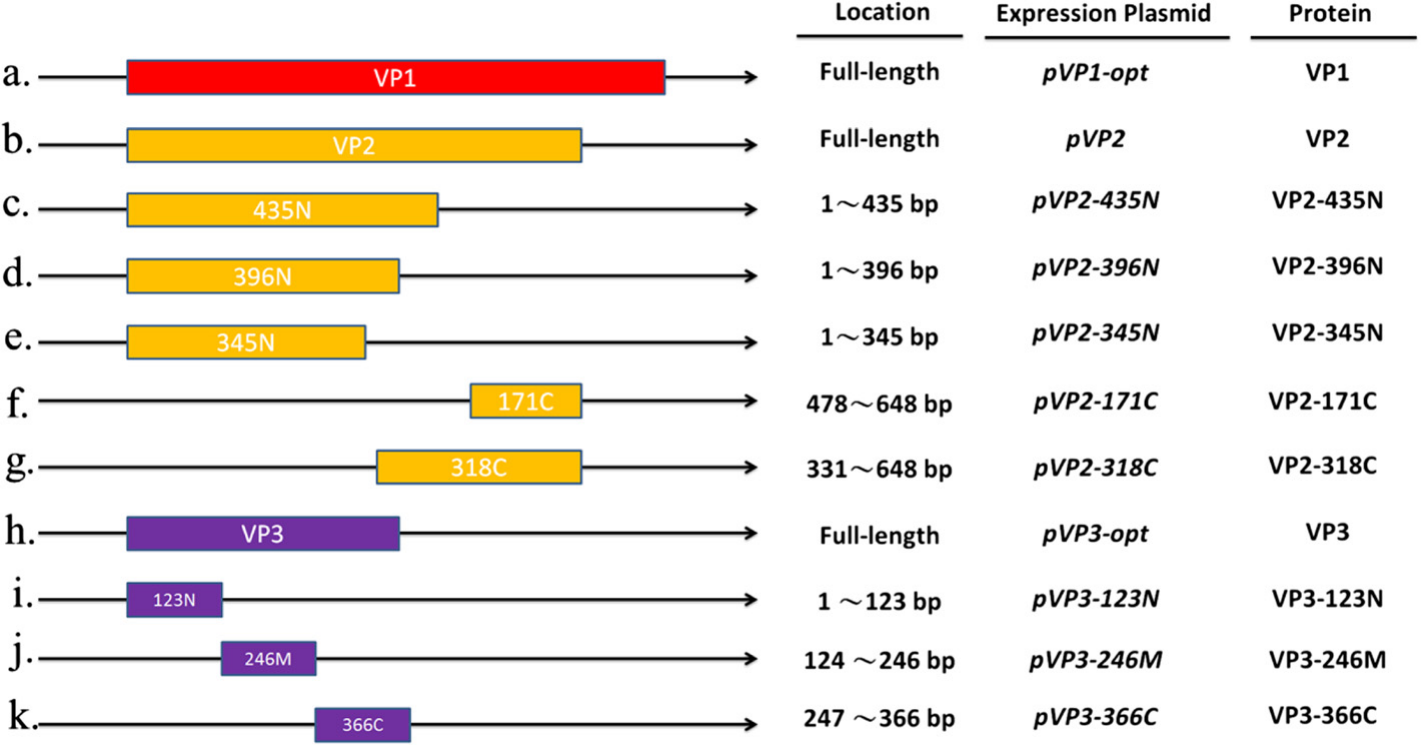
**The construction schemes used to produce the plasmids used for protein expression in this study.** The full and subunit viral protein genes of CAV, which are represented as different color boxes, were cloned into the pGEX-4T-1 expression vector, which has a *Tac* promoter and GST tag protein. VP1 (red box) and VP3 (purple box) means codon-optimized VP1 and VP3 protein genes that have been modified by rare codon optimization as described in previous reports [14,15].

**Figure 2:**
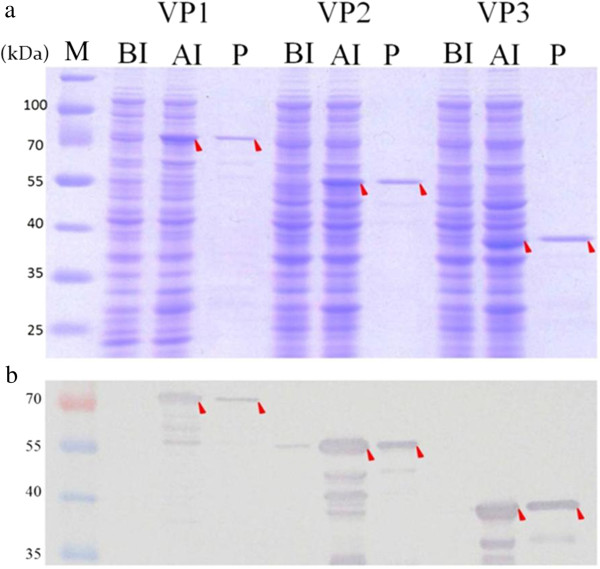
**The expression and purification of recombinant CAV viral proteins.** The three expression plasmids showed in Figure 1 were express in *E. coli* BL21 (DE3) and purified by GST affinity chromatography. The recombinant GST-tag proteins were separated by SDS-PAGE **(a)** and detected by Western-blotting **(b)**. Anti-GST tag monoclonal antibody was used to recognize the recombinant viral proteins. Lane M, pre-stained protein marker; lane BI, before IPTG induction; lane AI, after IPTG induction and cultivation for 4 hr; lane P, purified recombinant GST-tag protein. The red solid triangles pinpoint the various proteins.

**Figure 3:**
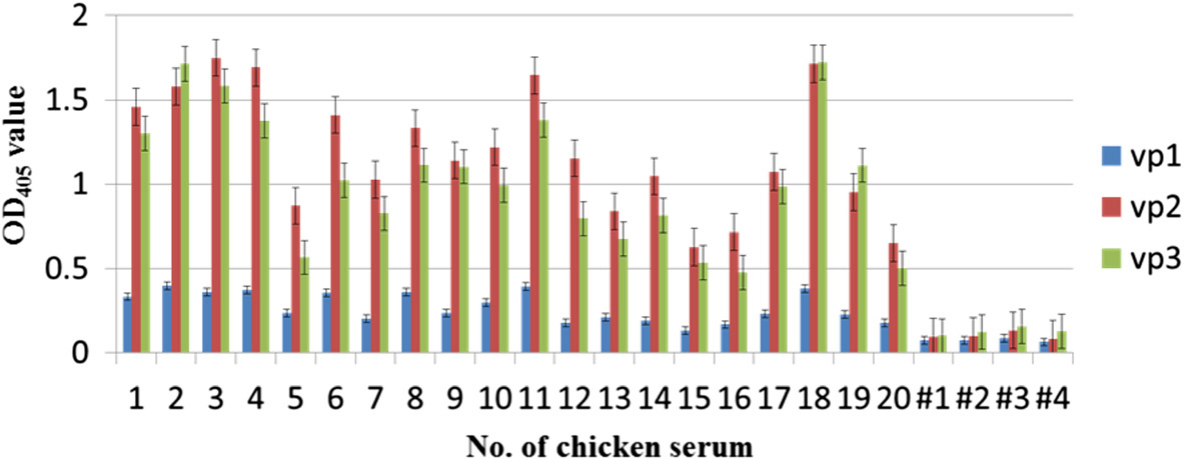
**Antigenicity of the purified recombinant GST-tag viral proteins using CAV-infected chicken sera.** Reactivity of twenty groups of CAV-infected positive chicken sera and four groups of negative sera (samples #1 to #4) with the recombinant protein was evaluated by indirect ELISA. These sera had been identified as negative or positive using a commercial ELISA kit purchased from the IDEXX laboratory Inc.

In order to express CAV VP1, VP2 and VP3 proteins as antigens for antigenic characterization, the respective recombinant constructs, pVP1-opt, pVP2 and pVP3-opt, harboring the cDNA of VP1, VP2 and VP3, were transformed into *E. coli* BL21 (DE3) and *E. coli* BL21(DE3)-pLysS (Figure 1, panel a, b, h). These strains were the used to express the various proteins after 4 hrs. induction with IPTG. As illustrated in Figure 2, the VP1, VP2 and VP3 proteins were successfully expressed in *E. coli* and produced the correct size bands on coomassie blue gels; these proteins also were recognized by anti-GST tag antibodies (Figure 2a and b). The estimated molecular weight of the induced VP1, VP2 and VP3 fusion proteins were 78 kDa for VP1, 52 kDa for VP2 and 40 kDa for VP3 proteins, each of which includes 28 kDa of the fused GST tag. After affinity chromatography purification, the purified recombinant VP1, VP2 and VP3 proteins were determined by SDS-PAGE and Western-blot analysis (panel P of Figure 2a). The antigenicities of the purified VP1, VP2 and VP3 proteins were then explored by ELISA as shown in Figure 3. The results were obtained as OD_405_ values. The OD_405_ values for the CAV-positive sera showed that the VP2 and VP3 protein had higher reactivity than VP1 and, furthermore, there were significant differences between the OD values for the positive and negative sera (Figure 3). The findings indicate that the VP2 and VP3 proteins exhibit better antigenicity than the VP1 protein when CAV-positive sera were used to recognize the antigen. Thus, for the development of an diagnostic kit, the VP2 and VP3 protein seem to have considerably better antigenic characteristics and sero-diagnostic potential.

### Screening and antigenic characterization against chicken sera of VP2 and VP3 subunit proteins

**Figure 4:**
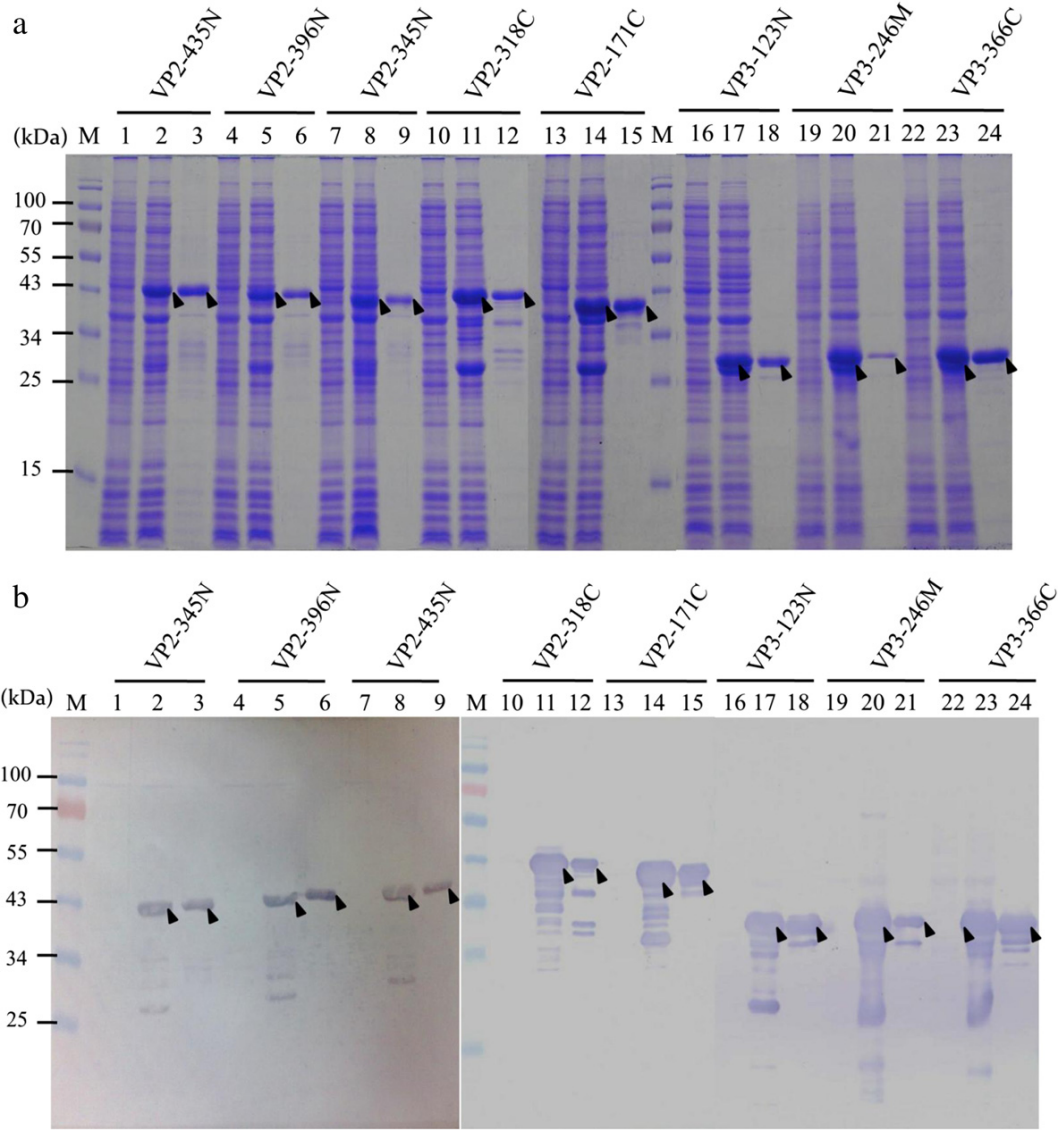
**The expression and purification results for the five recombinant VP2 and three recombinant VP3 subunit proteins.** All subunit proteins were purified by GST affinity chromatography after expression in *E. coli* BL21 (DE3). The presence of the protein was detected by SDS-PAGE **(a)** and by Western-blotting **(b)**. Anti-GST tag monoclonal antibody was used to recognize the VP2 and VP3 subunit proteins when the various different expression plasmids, as described in Figure 1, were used. Lane M, pre-stained protein marker; lane 1, 4, 7, 10, 13, 16, 19 and 22, before IPTG induction; lane 2, 5, 8, 11, 14, 17, 20 and 23, after IPTG induction and 4 h cultivation; lane 3, 6, 9, 12, 15, 18, 21 and, 24 after purification by GST affinity chromatography. The solid triangles pinpoint the expressed and purified VP2 and VP3 subunit proteins, respectively.

**Figure 5:**
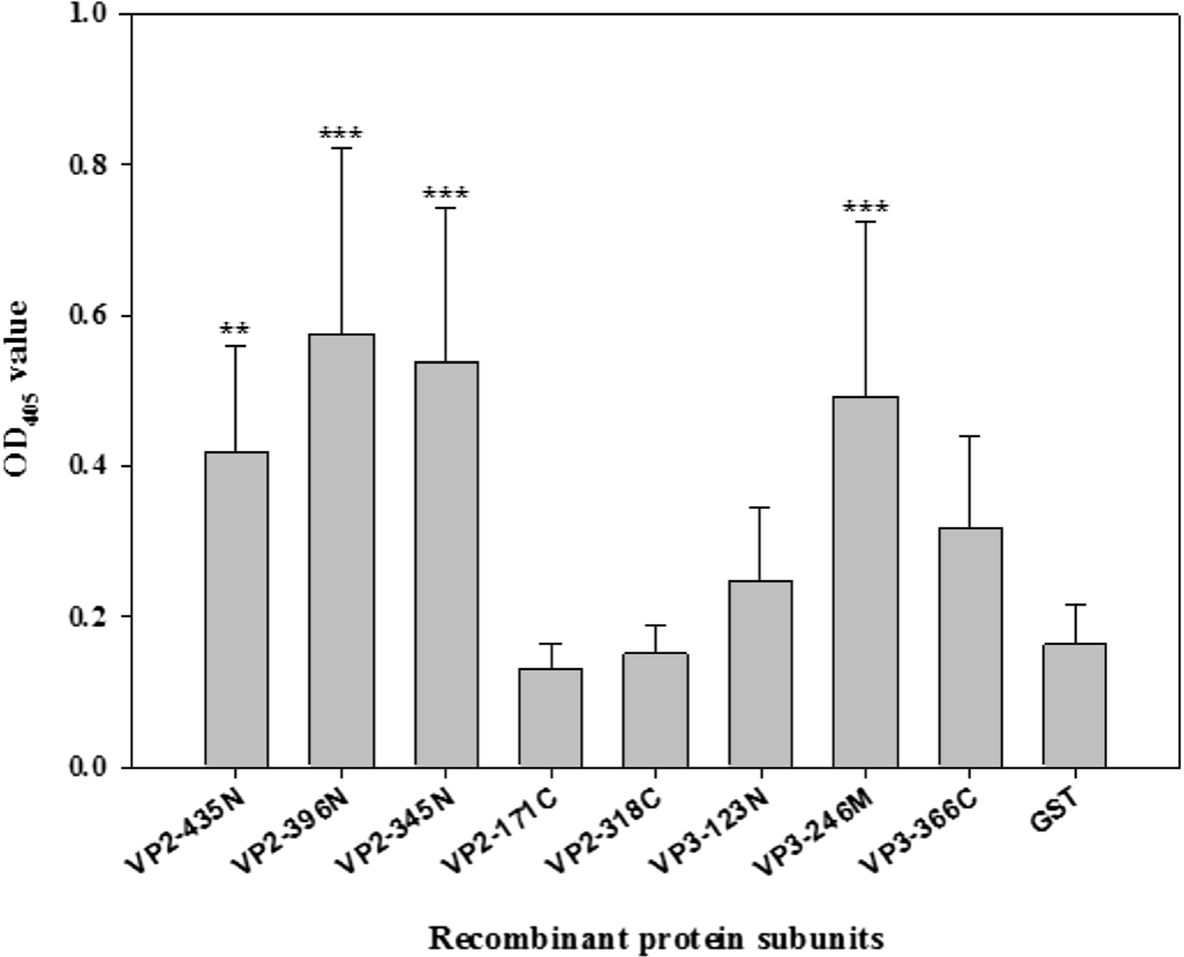
**The indirect ELISA results obtained using the various recombinant subunit proteins.** The five purified GST-fused VP2 subunits (VP2-435N, VP2-345N, VP2-396N, VP2-171C and VP2-318C) and the three VP3 subunit proteins (VP3-123N, VP3-246M and VP3-366C) were reacted with sera from 20 CAV-positive chicken. The mean of the optical density values at 405 nm (OD_405_) per subunit-base ELISA were determined from experimental triplicates, and the error bars indicate the standard errors of the means. The statistical significant were calculated by Scheffe’s S method which compared the OD_405_ value of VP2 or VP3 protein subunits to the value of GST recombinant protein. ***: *p*<0.001; **: *p*<0.01.

To optimally establish a CAV viral subunit protein-based indirect ELISA system, five recombinant VP2 subunit proteins and three VP3 subunit proteins were produced and expressed in an *E. coli* expression system; then their reactivity against CAV specific-positive sera was analyzed. Schematic diagrams of the VP2 and VP3 subunit proteins are presented in Figure 1, three consist of VP2 C-terminus deletion mutant proteins (VP2-435N, VP2-396N and VP2-345N), two consist of VP2 N-terminus deletion mutant proteins (VP2-171C and VP2-318C), one is a VP3 C-terminus deletion protein, another is a VP3 internal domain protein and the last is a N-terminus deletion protein (VP3-123N, VP3-246M and VP3-366C, respectively). After construction and expression in *E. coli*, the SDS-PAGE and Western blot results confirmed that all of the VP2 and VP3 subunit proteins were successfully expressed in *E. coli* (Figure 4a, b). The five VP2 subunit proteins (VP2-435N, VP2-396N, VP2-345N, VP2-171C and VP2-318C) and three VP3 subunit proteins (VP3-123N, VP3-246M and VP3-366C) after expression were purified and then evaluated to measure their reactivity against CAV-negative and CAV-positive specific chicken sera using an ELISA approach. All three N-terminal containing VP2 subunit proteins, VP2-435N, VP2-396N and VP2-345N, showed better reactivity compared to the two C-terminal containing VP2 subunit proteins, VP2-171C and VP2-318C, using 200 fold diluted CAV specific-positive chicken sera (Figure 5). Moreover, when these three N-terminal containing VP2 subunit proteins were compared against each other, the antigenic reactivity of VP2-345N and VP2-396N were higher than that of VP2-435N (*p*<0.001). In contrast, among the three VP3 subunit proteins, only subunit VP3-246M was demonstrated to be highly antigenic and show a significant difference in OD_405_ value between the CAV specific-positive (*p*<0.001) (Figure 5) (Additional file 1: Figure S2). Thus, based on the above findings, only two purified VP2 subunit proteins, 345N and 396N, together with one VP3 subunit protein, 246M, show characteristics suggesting that they contain major immunorelevant domains from either VP2 or VP3. Such subunit proteins have high potential as coating antigens for use in the sero-diagnosis of CAV infection or in other immunological applications.

### Diagnostic application of an ELISA based system using VP2-345N, VP2-396N and VP3-246M for the detection of CAV infection

**Figure 6:**
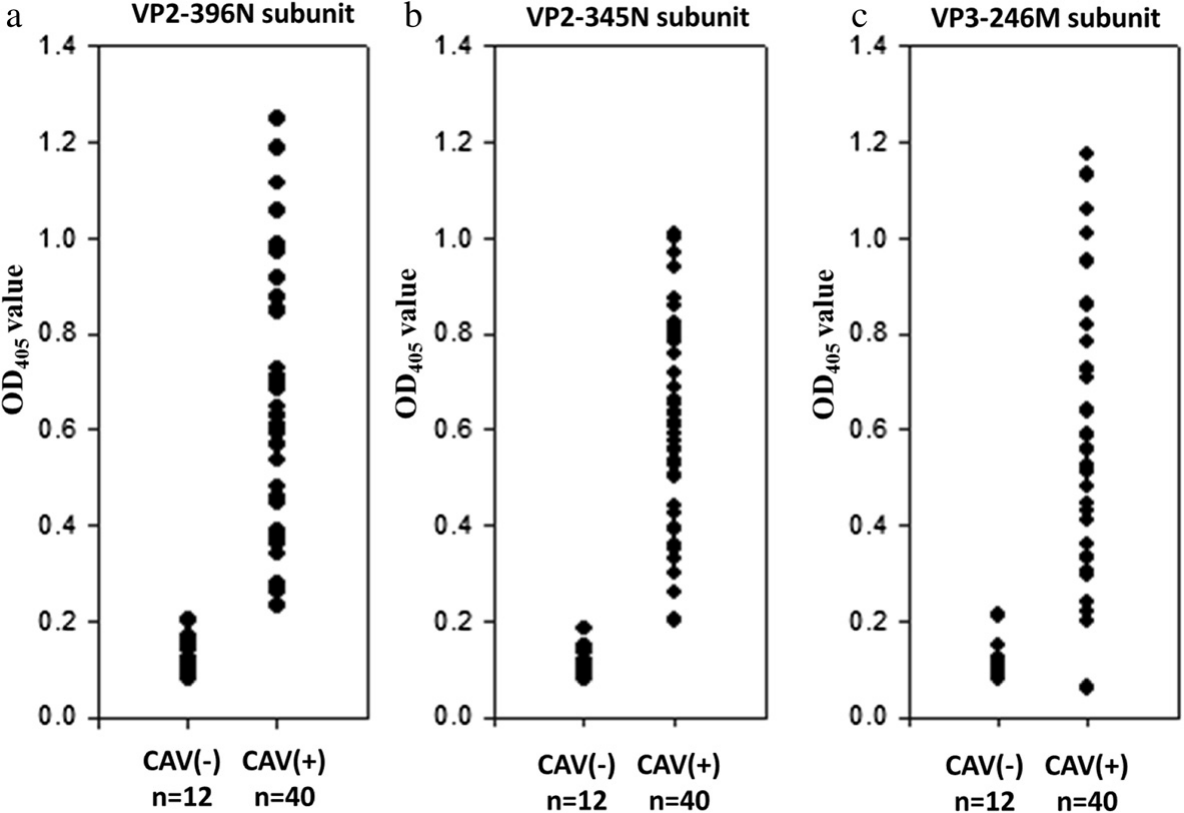
**Dot plot diagrams representing the reactivities of chicken serum with the three recombinant subunits of VP2 and VP3 proteins as determined by the indirect ELISA.** Forty CAV-positive and twelve CAV-negative chicken sera samples were used to obtain reactivity against the subunits VP2-396N **(a)**, VP2-345N **(b)** and VP3-246M **(c)**. The OD values at 405 nm (OD_405_) were obtained by recombinant protein-based ELISA.

To evaluate the possible clinical application of the VP2-345N, VP2-396N and VP3-246M proteins for the diagnosis of CAV infection, these three CAV viral subunit proteins were used in a CAV infection testing system. As shown as in Figure 6, when 40 CAV specific-positive and 12 CAV-negative chicken sera were analyzed using this approach, high antigenicity was demonstrated and there were significant differences in OD_405_ values between the CAV specific-positive and negative sera (*p*<0.01) using VP2-345N, VP2-396N and VP3-246M as coating antigens (Figure 6a-c). This indicated that the VP2-345N, VP2-396N and VP3-246M subunit proteins are highly antigenic and are able to discriminate chicken sera that have been CAV infected from those that have not been infected. Next, the 12 CAV negative chicken sera were tested against VP2-345N, VP2-396N and VP3-246M and the individual OD_405_ value as shown in Figure 6a-c were averaged to determine a positive threshold. Positive/negative cut-off values were determined as either the mean plus two standard deviations (mean+2SD) or as the mean plus three standard deviations (mean+3SD) (Additional file 1: Table S1). Using the mean+2SD as the cut-off value, the VP2-345N, VP2-396N and VP3-246M based ELISAs all produced 91.7% specificity. When the mean+3SD was used as the cut-off value, the specificities of the VP2-345N, VP2-396N and VP3-246M based ELISAs were all raised to 100% (Table 1). The sensitivities of the VP2-396N, VP2-345N and VP3-246M based ELISA were also determined and found to be 100% (40/40×100%) for VP2-396N, 100% (40/40×100%) for VP2-345N and 90% (36/40×100%) for VP3-246M, when the cut-off values were set at the mean+2SD. (Table 1). However, the sensitivities all decreased when the mean+3SD was applied as the cut-off value. In the latter circumstances, the sensitivities of the VP2-345N, VP2-396N and VP3-246M based ELISAs were determined to be 97.5% (38/40×100%), 97.5% (38/40×100%) and 85% (34/40×100%) (Table 1), respectively.

**Table 1:** Summary of specificities and sensitivities of three protein subunits

**Indirect ELISA to viral protein subunits**	**Cut-off with mean + 2 S.D.**			**Cut 0ff with mean + 3 S.D.**		
	**OD value**	**Specificity**^**a**^**(%)**	**Sensitivity**^**b**^**(%)**	**OD value**	**Specificity**^**a**^**(%)**	**Sensitivity**^**b**^**(%)**
VP2-396N	0.200	91.7 (11/12)	100 (40/40)	0.236	100 (12/12)	97.5 (39/40)
VP2-345N	0.185	91.7 (11/12)	100 (40/40)	0.217	100 (12/12)	97.5 (39/40)
VP3-246M	0.195	91.7 (11/12)	90 (36/40)	0.232	100 (12/12)	85 (34/40)

^a^: The percentage of specificity was determined from the numbers of OD_405_ value below cut-off values in the total ELISA results with 12 negative CAV-infected sera.

^b^: The percentage of sensitivity was determined from the numbers of OD_405_ value higher cut-off values in the total ELISA results with 40 positive CAV-infected sera.

## Discussion

In this study, we have successfully produced in *E. coli* VP1 protein, VP2 protein, VP3 protein as well as a series of VP2 and VP3 subunit proteins in order to evaluate their antigenicity with respect to specific CAV-positive chicken sera. After assessment of their antigenicity against chicken sera, the VP2 and VP3 proteins were demonstrated to have higher antigenicity than the VP1 protein. In previous reports, VP1 and VP2 protein have been shown to elicit virus neutralizing antibodies in the host [9]. Thus, VP1 and VP2 were thought to be good candidates as immunogens for vaccine development or diagnostic application. However, the present antigenic analysis indicates that CAV VP1 protein may not be that useful in serological tests. During CAV infection, VP2 and VP3 are detected in chicken cells within 12 hours post-infection, while VP1 is only detected at 24 hours post-infection [7]. This phenomenon indicates that the titers of antibodies against CAV viral proteins produced in the chicken sera might have different levels that correspond to either exposure time or to the amount of viral protein present in the infected chicken. At present, there are no reports available that describe the IgG titer profiles in the chicken sera against the VP1, VP2 and VP3 proteins. Thus, a lower titer of anti-VP1 IgG in chicken sera might be present and this would result in a lower antigenicity when tested by ELISA.

Using a subunit protein rather than an intact virion as coating antigen during the development of serological test has some advantages; these include cost-effectiveness, time-saving and ease of antigen production. Indeed, currently, the greatest problem when developing a serological assay is identifying, obtaining and preparing a suitable antigen. This is especially true if intact virion or viral protein/antigen purified from virus-infected tissue or from cell culture is used. This is because these require tedious processing, including the concentration of supernatant from infected culture medium by continuous zonal centrifugation or by PEG precipitation. Moreover, the source of coating antigen is a crucial consideration when performing large-scaled antigen production. Therefore, using a recombinant subunit protein for serodiagnosis would be much simpler. Up to the present, there have been no reports describing post-translational modification of the native CAV VP1, VP2 and VP3 proteins. Based on this, in the present study, we used a prokaryotic expression system to produce recombinant CAV antigens. By direct engineering of the VP2 and VP3 proteins it was possible to product truncated subunit proteins that are more convenient and seem to be more suitable and more efficient than proteins produced by an insect-baculovirus system or by a transgenic plant approach [9,12,18]. When the productivities in *E. coli* of the recombinant VP2 and VP3 subunit proteins were evaluated, the highest expression levels of the VP2-345N, VP2-396N and VP3-246M proteins, both soluble and insoluble, in *E. coli* whole cell lysate after IPTG induction for 4 hrs. were determined to be 432.6 mg/L, 334 mg/L and 786.2 mg/L (Additional file 1: Figure S1A), respectively. In addition, a growth profile analysis of these three *E. coli* strains showed that the VP3-246M expressing strain had the fastest growth rate at 4 hours cultivation after IPTG induction when the three recombinant strains were compared (Additional file 1: Figure S1B). The growth profiles of the VP2-345N and VP2-396N expressing strains in *E. coli* were almost identical. These findings suggest that production of the VP3-246M protein in *E. coli* will be the most efficient.

Using an antigenic peptide prediction bioinformatics software program (http://imed.med.ucm.es/Tools/antigenic.pl), the antigenic propensities of VP2 and VP3 were analyzed (data not shown). Seven antigenic determinants were predicted to be present in the VP2 protein and these spanned amino acid residues 29-35 (AQGQVIS), 56-62 (KFTAVGN), 77-97 (NHSIAVWLRECSRSHAKICNC), 104-110 (WFQECAG), 119-132 (SLEEAILRPLRVQS), 137-145 (RKLDYHYSQ) and 195-204 (DSGIVDELLG). Only four antigenic determinants were predicted to be present in the VP3 protein and these peptides spanned amino acid residues 11-18 (GPSTVFRP), 24-32 (PLETPHCRE), 34-51 (RIGIAGITITLSLCGCAN) and 86-94 (KKRSCDPSE). Thus VP2-435N consists of at least six antigenic determinants. However, the antigenic reactivity of VP2-435N was lower than that of VP2-345N and VP2-396N (Figure 5). The result of these expressed recombinant proteins might not be correctly folfed to that of the native protein; in such circumstances all of the antigenic regions of the subunit protein might not be exposed completely on the protein surface. Moreover, higher immunoreactivity was observed with the N-terminal containing VP2 subunit proteins, which suggests that there are functional antigenic sites located within the N-terminal region of this protein. In a previous study, a comparison of the amino acid sequences of the CAV VP2 protein across the different CAV isolates available in the GenBank demonstrated extremely high identity between these isolates [19]. In such circumstances, N-terminal containing VP2 subunit proteins containing discriminating immunorelevant epitopes should be useful when developing subunit protein-based ELISAs for the detection of CAV specific antibodies.

VP3-123N has at three predicted antigenic determinants, which contrast with VP3-246M, which has only one predicted antigenic determinant; nevertheless, the antigenic reactivity of VP3-246M was found to be higher than that of the other VP3 subunit proteins, VP3-123N and VP3-366C. This may indicate that the central domain of the VP3 protein, VP3-246M, is a major factor in antigenic recognition. Taking the above results together, the findings suggest that VP2-345N, VP2-396N, VP2-435N and VP3-246M all seems to be structurally and antigenically very similar to their native protein, at least to some degree (Additional file 1: Figure S3). Consequently, our findings demonstrate that the above VP2 and VP3 subunit proteins are likely to be good candidates for sero-diagnostic application in the future.

## Conclusions

This is first report to describe the expression, purification and systematically antigenic characterization of CAV viral proteins. Engineered subunit proteins, VP2-345N, VP2-396N and VP3-246M, are likely to be more useful in sero-diagnostic kits for CAV antibodies detection than the full-length native VP2 and VP3 proteins. This is because they should be cost-effectiveness as well as having high antigenicity; they thus ought to be useful as coating antigens when used in immune-enzymatic systems.

## Methods

### Bacterial strains and inoculation

Two *E. coli* strains, BL21 (DE3) and BL21(DE3)-pLysS were purchased from Invitrogen Life Technologies (Carlsbad, CA) and Stratagene (La Jolla, CA), respectively. Strain activation was performed using 10 ml of LB medium in 50 ml flasks by growing overnight at 37°C. The overnight culture were then inoculated into 50 ml LB medium and grown at 37°C for 3 h by which time the optical density of the culture had reach 0.5 of OD_600_ and could be used for competent cell preparation and protein expression.

### Construction of the expression vectors

The entire length of the cDNA, Opt-VP1, which has undergone codon optimization of the N-terminus of the CAV VP1 capsid protein, was ligated into pGEX-4T-1 to give pVP1-opt (Figure 1, panel a); this was then used as described in a previous study [15]. The other two constructs, pVP2 and pVP3-opt, which harbor the full-length cDNAs of the CAV VP2 and VP3 genes respectively, were subcloned from the plasmids pET28a-VP2 [13] and pVI127-VP3 [14] into the plasmid pGEX-4T-1 using the cloning sites *EcoR*I and *Xho*I (Figure 1, panel b and c). In order to create the various VP2 subunit proteins, the expression plasmids, pVP2-435N, pVP2-396N, pVP2-345N, pVP2-171C and pVP2-318C, were constructed as described below. Specifically, all of the DNA fragments for the VP2 subunit proteins were amplified by PCR using high fidelity Platinum *Taq* polymerase (Invitrogen) with pVP2 as the target; the primers are summarized in Table 2. In order to create the VP3 subunit proteins, the three recombinant plasmids, pVP3-123N, pVP3-246M and pVP3-366C, were created following the same gene cloning procedures. The primers used for the construction of the VP3 subunit DNA fragments are also listed in Table 2. All of the constructed recombinant plasmids were transformed into One Shot® Top10 (Invitrogen, CA) chemically competent *E. coli* for maintenance of the recombinant plasmids and to allow protein expression. Transformants that contained a gene of the correct size by PCR were then checked using restriction enzyme digestion and DNA sequence analysis.

**Table 2:** The primers sequences used for insert DNA amplification of the VP2 and VP3 subunit genes by PCR

**Subunit**	**Primer name**	**Type**	**Sequence (5’-3’)**
**VP2-345N**	VP2-N*EcoR*I	Forward	TGGAATTCATGCACGGGAACGGCGGA
	VP2-115C del *Xho*I	Reverse	TCCTCGAGTGATCGGTCCTCAAGT
**VP2-396N**	VP2-N*EcoR*I	Forward	TGGAATTCATGCACGGGAACGGCGGA
	VP2-131C del *Xho*I	Reverse	TCCTCGAGACCCTGTACTCGGAG
**VP2-435N**	VP2-N*EcoR*I	Forward	TGGAATTCATGCACGGGAACGGCGGA
	VP2-144C del *Xho*I	Reverse	TCCTCGAGCTGGGAGTAGTGGTAATC
**VP2-171C**	VP2-170N del *EcoR*I	Forward	AGGAATTCATGGACGAGCTCGCAGAC
	VP2-C*Xho*I	Reverse	TCCTCGAGCACTATACGTACCGG
**VP2-318C**	VP2-111N del *EcoR*I	Forward	TGGAATTCATGGAGGACCGATCAACC
	VP2-C*Xho*I	Reverse	TCCTCGAGCACTATACGTACCGG
**VP3-123N**	VP3-N *EcoR*I	Forward	TGGAATTCATGAACGCTCTGCAGGAA
	VP3-243C del *Xho*I	Reverse	TCCTCGAGTCAAGTAATGCCAGCGAT
**VP3-246M**	VP3-123N del *EcoR*I	Forward	TGGAATTCATGATTACTCTGTCCCTG
	VP3-120C del *Xho*I	Reverse	TCCTCGAGTCATTTCGGCTGATCAGT
**VP3-366C**	VP3-246N del *EcoR*I	Forward	TGGAATTCATGCCTCCGTCCAAGAAA
	VP3-C *Xho*I	Reverse	TCCTCGAGTTACAGGCGGATGCAACG

Primer names describe the annealing locations of target genes and the restriction enzyme names used in expression plasmid cloning.

### Expression and production of the CAV viral proteins, VP1, VP2 and VP3 as well as the truncated VP2/VP3 subunit proteins in the recombinant *E. coli*

The recombinant *E. coli* strains BL-21 (DE3) and BL21(DE3)-pLysS containing the constructs as shown in Figures 1 and 4(a) were used for protein induction and expression. The recombinant strains were grown overnight in LB medium in the presence of ampicillin (50 μg/mL) at 37°C. Then 0.5 mL of overnight culture was inoculated into 50 mL LB medium and grown at 37°C for 3 hrs. by which time the optical density of culture had reached 0.5. At this point, isopropyl-β-D-thiogalactopyronoside (IPTG) at 0.1 mM was added to the culture to induce protein expression, which continued for 4 hrs. The presence of expressed recombinant proteins were examined by 12.5% SDS-PAGE followed by Western blotting using monoclonal anti-GST antibody or CAV-infected positive sera.

### Purification of recombinant CAV viral proteins using GST affinity chromatography

To purify the recombinant CAV viral proteins, both VP1, VP2 and VP3 as well as the truncated VP2 or VP3 subunit proteins, the cells were spun down from 50 mL of culture supernatant and resuspended in GST resin binding buffer (140 mM NaCl, 2.7 mM KCl, 10 mM Na_2_HPO_4_, 1.8 mM KH_2_PO_4_, pH 7.3). The harvested cells were disrupted and prepared as described previously [15]. The resulting cell supernatant was loaded onto a GSTrap FF affinity column (GE healthcare, Piscataway, NJ) to allow protein purification using the optical unit of a liquid chromatography system (AKTAprime plus, GE Healthcare BioScience AB, Uppsala, Sweden). The operational conditions were the same as described in a previous study [13,15]. The total protein concentration of the collected eluent containing the recombinant CAV viral proteins was determined using a Micro BCA kit (Pierce, Rockford, IL) with bovine serum albumin as the reference protein. The purity of the protein sample was analyzed by 12.5% SDS-PAGE and Western-blotting with appropriate antibodies using fraction aliquots.

### ELISA assays

To compare the antigenicity of the *E. coli* expressed CAV viral proteins,VP1, VP2 and VP3 as well as the truncated VP2 or VP3 subunit proteins, ELISA assays were performed. The various purified recombinant CAV viral proteins after affinity GST-column purification were used as the coating antigen for the ELISAs. Briefly, same copy numbers per well of diluted VP1, VP2 and VP3 as well as the truncated VP2 or VP3 subunit proteins were used to coat a 96 wells plate for 1 h at 37°C. After washing, A 200 fold dilution of CAV-negative and CAV-positive specific chicken sera, which had been obtained from an experimental farm and had been identified as negative or positive using a commercial ELISA kit (purchased from the IDEXX laboratory Inc.), were added and reacted for another hour at 37°C. Subsequently, following washing, secondary antibodies (peroxidase conjugated affinipure mouse anti-chicken IgG; Jackson) were added and this was followed by color development as described in previously [14].

### Statistical analyses

Data are expressed as mean±standard deviation, each experiment was performed at least three independent times. The significant of difference between groups was determined using a Scheffe’s S method. Statistical probability of *p*<0.05 was defined statistically significant. Statistical analysis was performed using SigmaPlot.

## Competing interests

All authors declare no competing interests.

## Authors’ contributions

MSL participated in this study design, performed the experiments and in the writing of the manuscript. GHL performed the experiments, study design and participated in the construction of the plasmids. YYL participated in the experiments on protein antigenicity and GHL, CHH, and JHF participated in the protein purification step and performing ELISA assay. JTCT participated in the data analysis and the writing of the manuscript. HJC coordinated the study and participated in performing ELISA assay. All authors read and approved the final manuscript.

## Supplementary Material

Additional file 1 **Production profiles, indirect ELISA and antigenicity analysis of the *E. coli*-expressed VP2 and VP3 subunit proteins. Figure S1.** Production profiles and growth kinetics of the subunits VP2-396N, VP2-345N and VP3-246M proteins using the various *E. coli* strains. (A) The production profiles of the three *E. coli* strains expressing VP2-396N, VP2-345N and VP3-246M proteins are showed over a 4 h time course after IPTG induction. (B) Growth profiles of the three *E. coli* strains expressing VP2-396N, VP2-345N and VP3-246M proteins in LB medium post-induction. The protein quantity was determined before isolation. The protein concentration was quantified by measuring the intensity of the protein bands on Coomassie blue R250 stained gels using a densitometer. Also, this method was performed following our previously work [13-15]. **Figure S2.** The indirect ELISA results of the recombinant subunit proteins. Five purified GST-fused VP2 subunits (VP2-435N, VP2-345N, VP2-396N, VP2-171C and VP2-318C) and three purified GST-fused VP3 subunits (VP3-123N, VP3-246M and VP3-366C) were reacted with sera of 20 CAV-positive chicken or with 12 sera of healthy young chickens (#21 to #32). The mean of optical density values at 405 nm (OD_405_) per subunit-base ELISA were determined from experimental triplicates, and error bars indicated standard errors of the means. These sera had been all identified as negative or positive using a commercial ELISA kit purchased from the IDEXX Laboratory Inc. The sera used herein and Figure 3 were the same batch. The sample sizes of sera used in this figure was much more than the sera used in the Figure 3, especially negative CAV-infected sera. **Table S1.** The table represents the optical density values at 405 nm (OD_405_) which were used in determination of cut-off value to reactivity of indirect ELISA on CAV-negative chicken serum with three subunits. All CAV-negative chicken sera were identified using commercial ELISA kit purchased from IDEXX Laboratory Inc. **Figure S3.** The Western blot results of the three potential high antigenicity domains of VP2 and VP3. The CAV-positive serum used for the indirect ELISAs in this study were also used as the primary antibody for the Western blotting assays. Lane M, pre-stained protein marker; lane 1, VP2-345N; lane 2, VP2-396N; lane 3, VP3-246M. Bold triangles indicate the recombinant subunits. Click here for file
