# Supplementary material for: Expression and characterization of highly antigenic domains of chicken anemia virus viral VP2 and VP3 subunit proteins in a recombinant E. coli for sero-diagnostic applications
Source: BMC Vet Res. 2013 Aug 13;9:161. doi: 10.1186/1746-6148-9-161 (PMC3751357; doi:10.1186/1746-6148-9-161)
Supplement: Additional file 1 — Production profiles, indirect ELISA and antigenicity analysis of the E. coli-expressed VP2 and VP3 subunit proteins. Figure S1. Production profiles and growth kinetics of the subunits VP2-396N, VP2-345N and VP3-246M proteins using the various E. coli strains. (A) The production profiles of the three E. coli strains expressing VP2-396N, VP2-345N and VP3-246M proteins are showed over a 4 h time course after IPTG induction. (B) Growth profiles of the three E. coli strains expressing VP2-396N, VP2-345N and VP3-246M proteins in LB medium post-induction. The protein quantity was determined before isolation. The protein concentration was quantified by measuring the intensity of the protein bands on Coomassie blue R250 stained gels using a densitometer. Also, this method was performed following our previously work [13-15]. Figure S2. The indirect ELISA results of the recombinant subunit proteins. Five purified GST-fused VP2 subunits (VP2-435N, VP2-345N, VP2-396N, VP2-171C and VP2-318C) and three purified GST-fused VP3 subunits (VP3-123N, VP3-246M and VP3-366C) were reacted with sera of 20 CAV-positive chicken or with 12 sera of healthy young chickens (#21 to #32). The mean of optical density values at 405 nm (OD405) per subunit-base ELISA were determined from experimental triplicates, and error bars indicated standard errors of the means. These sera had been all identified as negative or positive using a commercial ELISA kit purchased from the IDEXX Laboratory Inc. The sera used herein and Figure 3 were the same batch. The sample sizes of sera used in this figure was much more than the sera used in the Figure 3, especially negative CAV-infected sera. Table S1. The table represents the optical density values at 405 nm (OD405) which were used in determination of cut-off value to reactivity of indirect ELISA on CAV-negative chicken serum with three subunits. All CAV-negative chicken sera were identified using commercial ELISA kit purchased from IDEXX Laboratory Inc. Figure S3. [file 1746-6148-9-161-S1.docx]

**Supplementary figures and table**


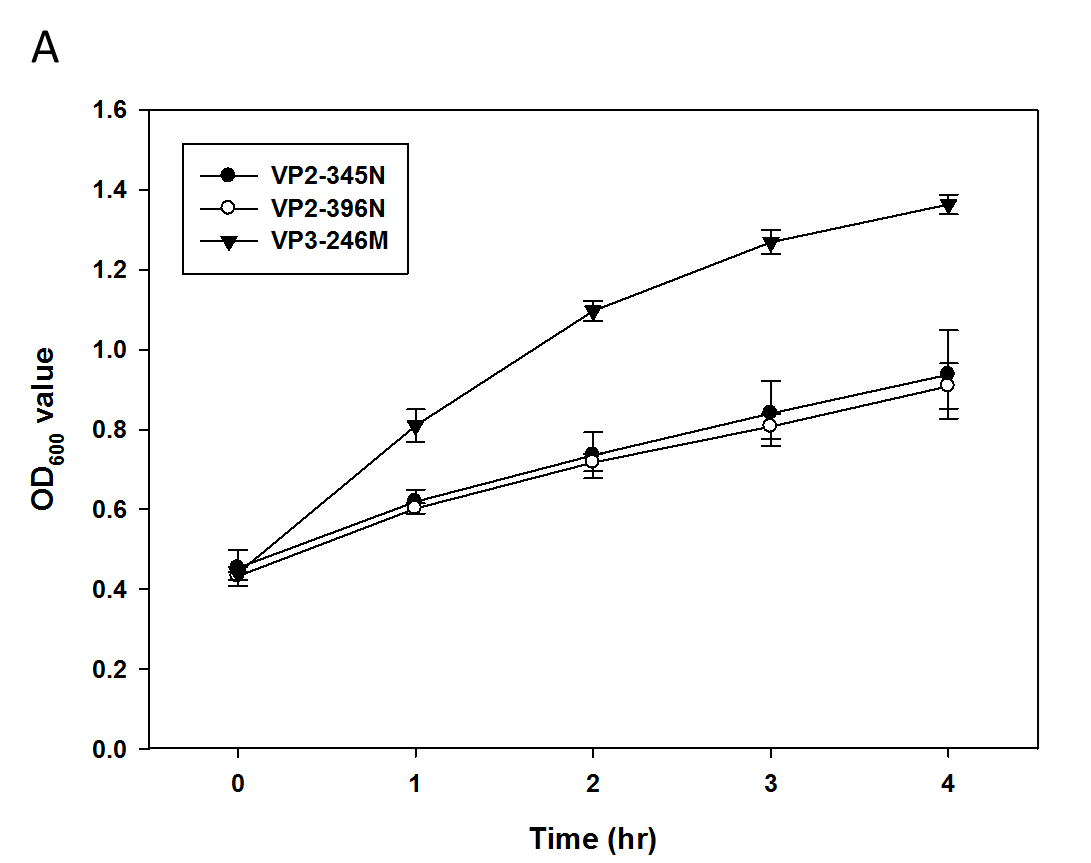

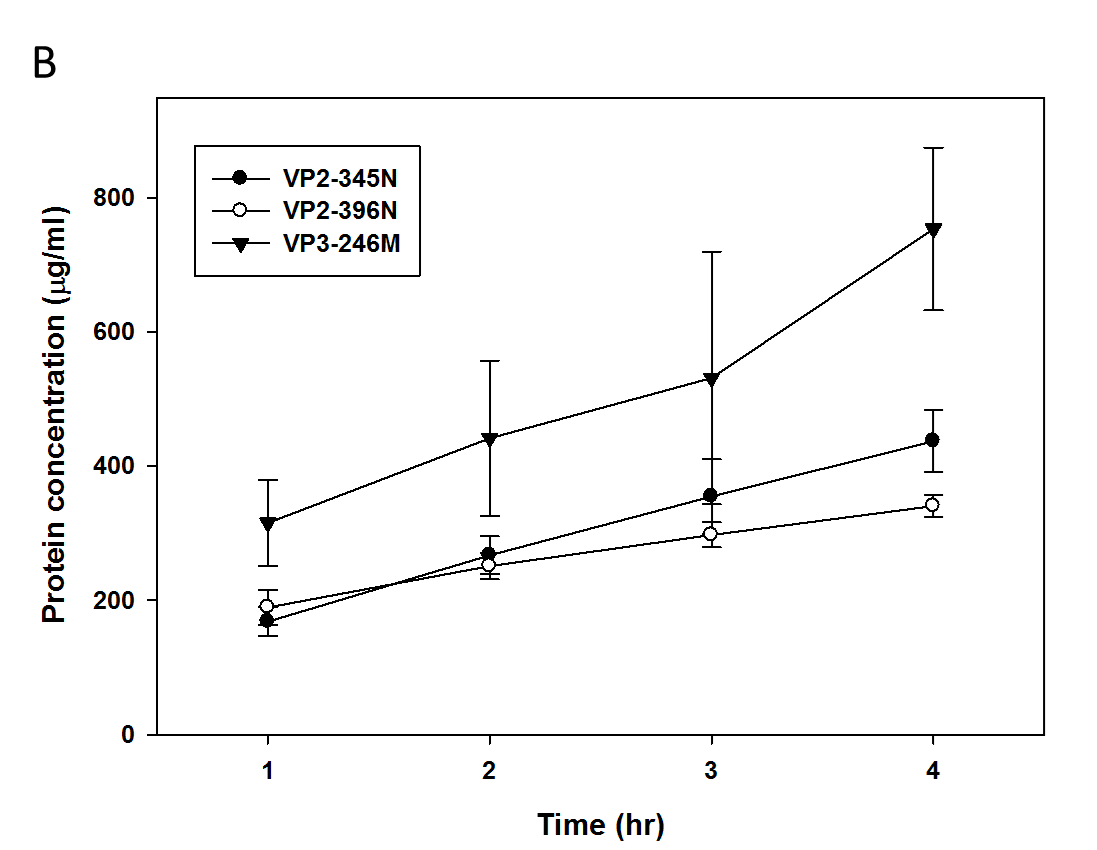


Supplementary figure 1. Production profiles and growth kinetics of the subunits VP2-396N, VP2-345N and VP3-246M proteins using the various *E. coli* strains. (A) The production profiles of the three *E. coli* strains expressing VP2-396N, VP2-345N and VP3-246M proteins are showed over a 4 h time course after IPTG induction. (B) Growth profiles of the three *E. coli* strains expressing VP2-396N, VP2-345N and VP3-246M proteins in LB medium post-induction. The protein quantity was determined before isolation. The protein concentration was quantified by measuring the intensity of the protein bands on Coomassie blue R250 stained gels using a densitometer. Also, this method was performed following our previously work [13, 14, 15].


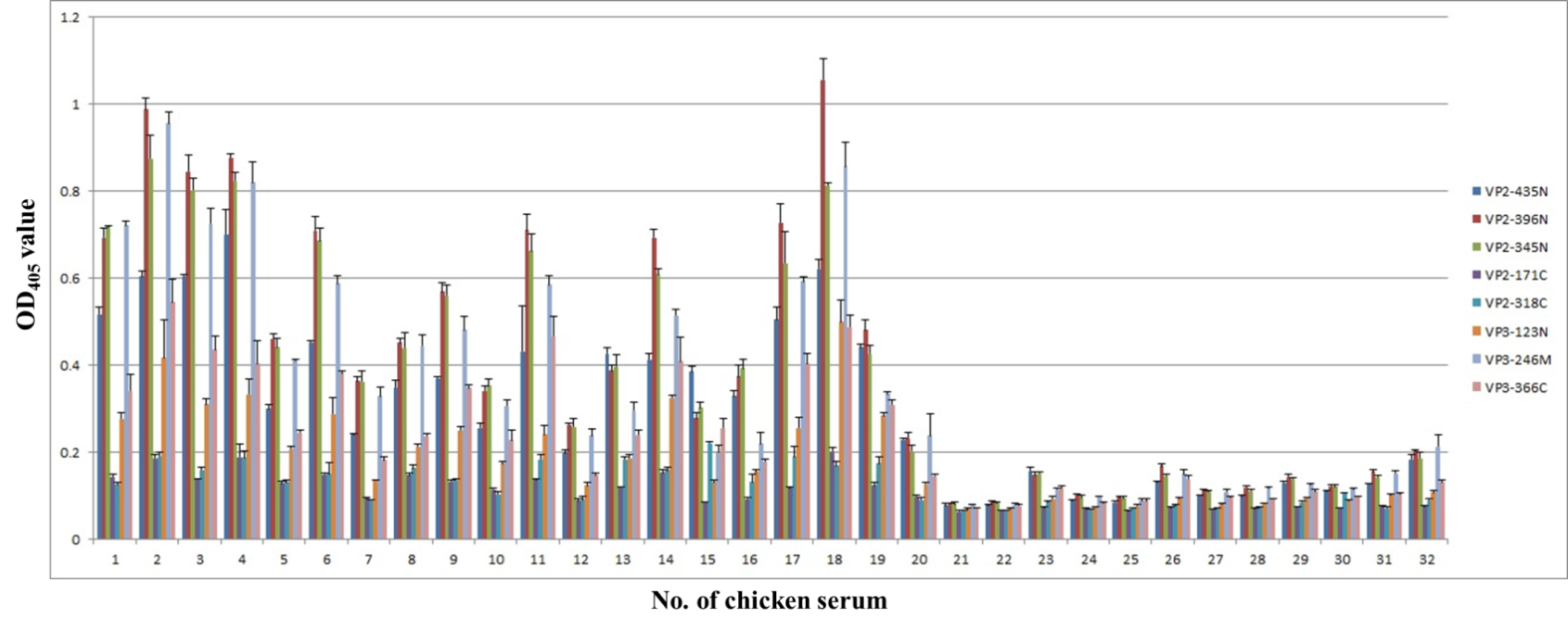


Supplementary figure 2. The indirect ELISA results of the recombinant subunit proteins. Five purified GST-fused VP2 subunits (VP2-435N, VP2-345N, VP2-396N, VP2-171C and VP2-316C) and three purified GST-fused VP3 subunits (VP3-123N, VP3-246M and VP3-366C) were reacted with sera of 20 CAV-positive chicken or with 12 sera of healthy young chickens (#21 to #32). The mean of optical density values at 405 nm (OD_405_) per subunit-base ELISA were determined from experimental triplicates, and error bars indicated standard errors of the means.These sera had been all identified as negative or positive using a commercial ELISA kit purchased from the IDEXX Laboratory Inc. The sera used herein and figure 3 were the same batch. The sample sizes of sera used in this figure was much more than the sera used in the figure 3, especially negative CAV-infected sera.

Supplementary Table 1. The table represents the optical density values at 405 nm (OD_405_) which were used in determination of cut-off value to reactivity of indirect ELISA on CAV-negative chicken serum with three subunits. All CAV-negative chicken sera were identified using commercial ELISA kit purchased from IDEXX Laboratory Inc.


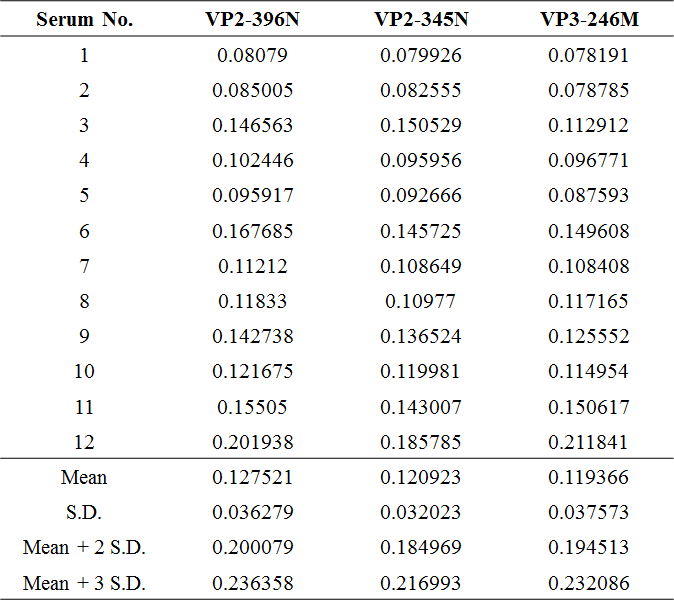

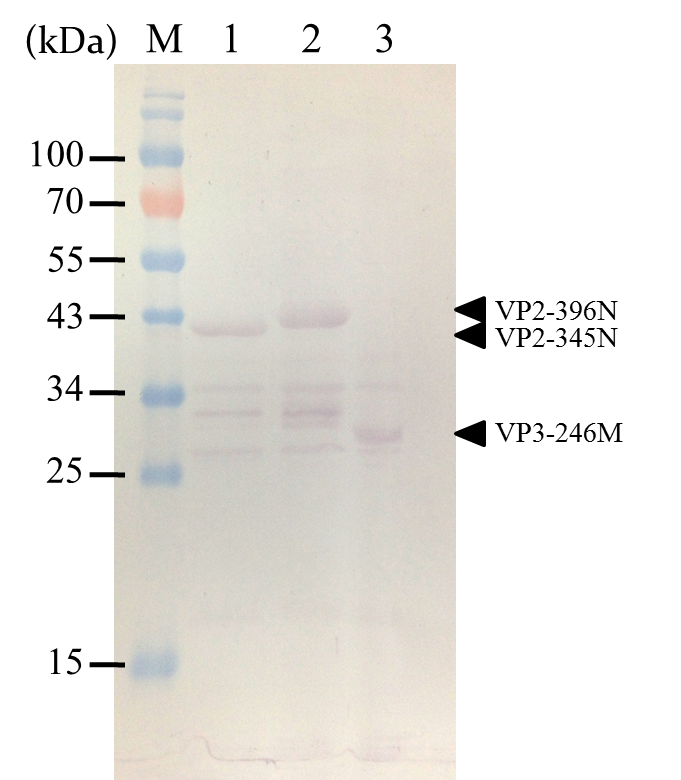


Supplementary figure 3. The Western blot results of the three potential high antigenicity domains of VP2 and VP3. The CAV-positive serum used for the indirect ELISAs in this study were also used as the primary antibody for the Western blotting assays. Lane M, pre-stained protein marker; lane 1, VP2-345N; lane 2, VP2-396N; lane 3, VP3-246M. Bold triangles indicate the recombinant subunits.
